# Arrangements for the Meeting at Fort Worth, April 22, 1890

**Published:** 1890-04

**Authors:** W. P. Burts

**Affiliations:** Chairman Arrangement Committee; Fort Worth


### ARRANGEMENTS.

Fort Worth, April 11, 1890.

*Dr. F. E. Daniel, Editor Daniel's Texas Medical Journal:*

The hotels claim that they are always full and are not much inclined to give rates. The Pickwick and Ellis will charge $2.50 per day, and the Mansion, $2.00; The Grand, $2.00; Ginochio's, $2.50. The meetings will be held in the Opera House.

Yours, very truly, W. P. Burts,

Chairman Arrangement Committee.

